# Point-of-Care Ultrasound for Earlier Detection of Pediatric Pneumonia

**DOI:** 10.5811/cpcem.7216

**Published:** 2024-07-18

**Authors:** John H. Priester, Prasanna Kumar, Jesse Naumann, Katherine Dolbec, Peter Weimersheimer, Christian D. Pulcini

**Affiliations:** * University of Vermont, Larner College of Medicine, Department of Emergency Medicine, Burlington, Vermont; † Brown University/Rhode Island Hospital, Department of Emergency Medicine, Providence, Rhode Island; ‡ University of Vermont, Larner College of Medicine, Department of Pediatrics, Burlington, Vermont

**Keywords:** *point-of-care ultrasound*, *pneumonia*, *lung ultrasound*, *chest radiograph*, *pediatrics*

## Abstract

**Case Presentation:**

An 8-month-old infant presented to a general emergency department with chief complaints of rhinorrhea, decreased activity, and fever. A point-of-care lung ultrasound (LUS) was performed at bedside with potential early findings of pneumonia. Based on these findings on LUS, a chest radiograph (CXR) was ordered and performed with no acute findings. He was discharged without antibiotics based on these findings; unfortunately, he returned two days later with worsening symptoms requiring chest tube placement, mechanical ventilation, and prolonged hospitalization for complicated bacterial pneumonia.

**Discussion:**

Pneumonia is a major cause of pediatric morbidity and mortality worldwide. Despite evidence supporting the utilization of LUS for the diagnosis of pediatric pneumonia, CXR remains the default imaging for clinical decision-making in most settings. In this case, earlier antibiotics and higher reliance on LUS for clinical decision-making may have prevented the morbidity associated with this hospitalization.

Population Health Research Capsule What do we already know about this clinical entity?

*Chest radiography is the conventional imaging modality of diagnosing pediatric pneumonia. However, lung ultrasound offers similar, if not better, sensitivity and specificity.*

What is the major impact of the image(s)?

*This case demonstrates how lung ultrasound can diagnose pediatric pneumonia earlier than chest radiography and should be the basis for clinical decision making.*

How might this improve emergency medicine practice?

*In the presence of abnormal lung ultrasound findings and normal chest radiography, emergency medicine physicians should strongly consider initiating treatment.*

## CASE PRESENTATION

**Image 1.**
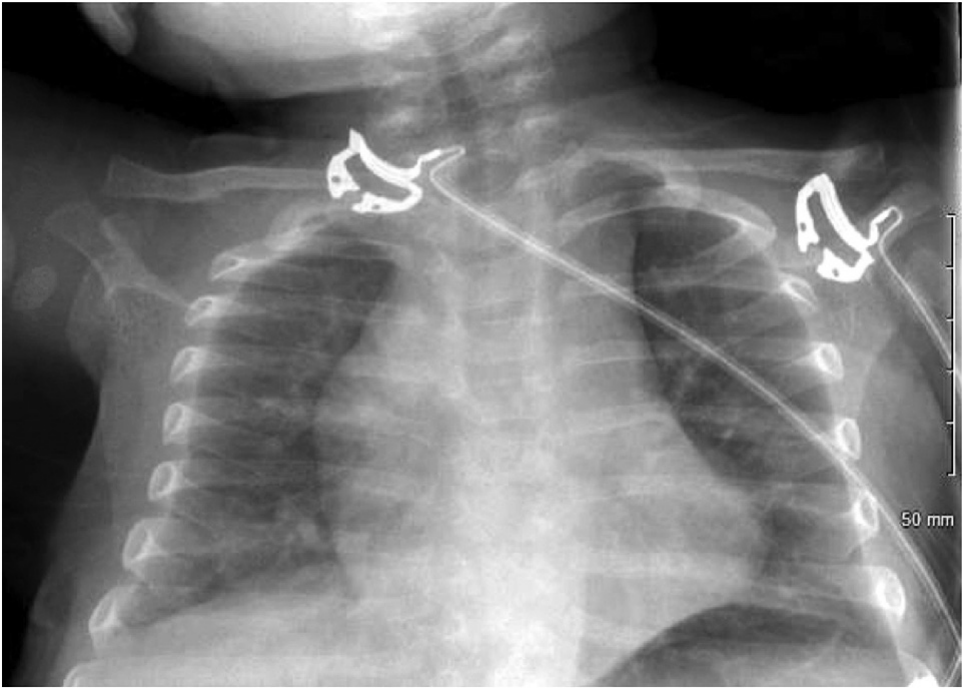
Chest radiograph from initial evaluation in the emergency department, without evidence of pneumonia.

**Image 2.**
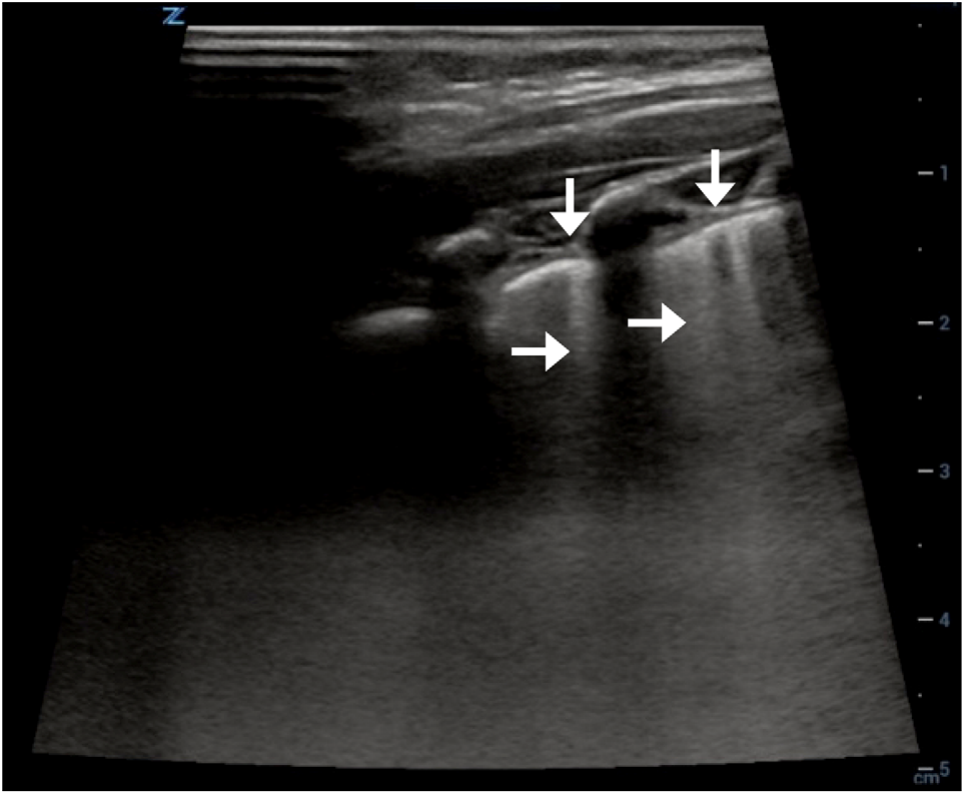
Focal pleural irregularities (vertical arrows) and B-lines (horizontal arrows) suggestive of pneumonia from right lung ultrasound performed on initial visit to the emergency department.

An 8-month-old infant presented to the emergency department (ED) with one week of rhinorrhea, decreased activity, and fever. Reported symptoms over the prior 24 hours included increased work of breathing, decreased oral intake, and fewer wet diapers. On arrival, physical exam findings included grunting, retractions, tachycardia, tachypnea, and fever of 39.3° Celsius. With antipyretics his respiratory symptoms improved. A chest radiograph (Image 1) was performed to follow up on an educational point-of-care lung ultrasound (LUS) (Image 2), which was suggestive of early pneumonia. He was discharged home without antibiotics given his negative chest radiograph (CXR) (Image 1).

**Image 3.**
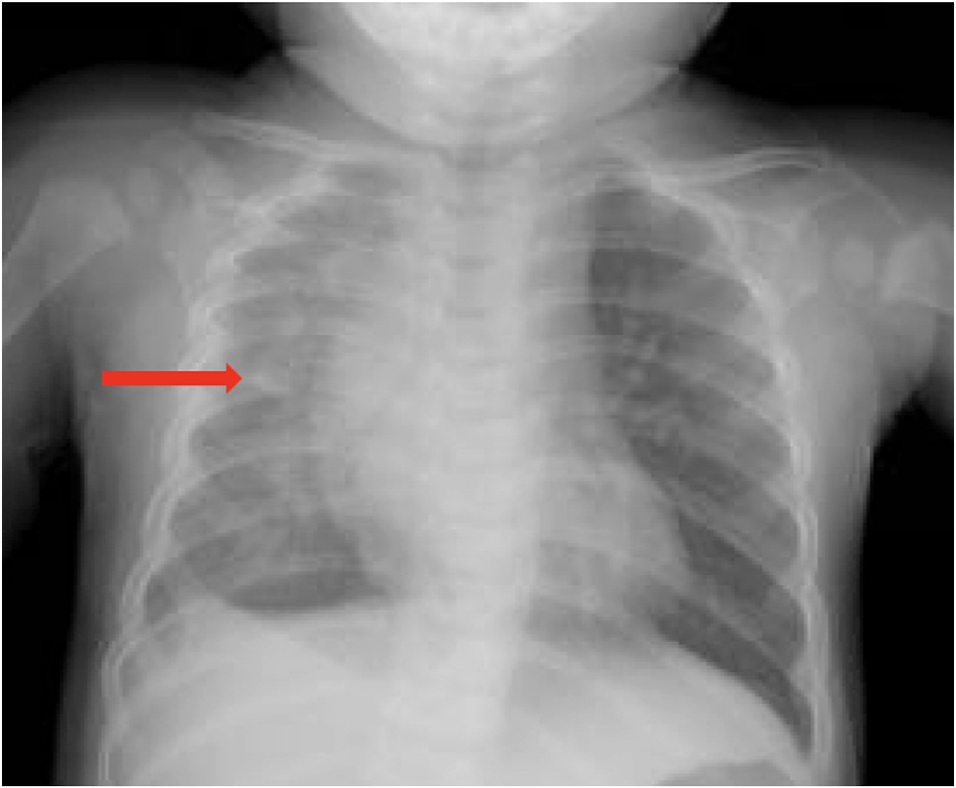
Chest radiograph on day 3, following return to the emergency department. Right lung consolidation suggestive of pneumonia is visible.

He unfortunately returned two days later with continuing fever, decreased oral intake, and emesis. Repeat radiography demonstrated right lung opacity, and he was diagnosed with methicillin-sensitive *Staphylococcus aureus* community-acquired pneumonia with empyema (Image 3). He was admitted and treated for pneumonia, and his course was complicated by acute hypoxic respiratory failure and empyema, necessitating mechanical ventilation and chest tube placement. He was discharged on hospital day 21.

## DISCUSSION

Community-acquired pneumonia is a major cause of pediatric morbidity and mortality.^1^ Our case adds to the current literature supporting LUS as superior in identifying pediatric pneumonia compared to CXR, yet CXR remains the most common modality used for clinical decision-making in this population in the ED.^2^ Previous research has compared LUS to CXR in the diagnosis of pneumonia in children with sensitivity and specificity of LUS as high as 96% and 93%, respectively.^2^ Further, up to 28% of lesions in pediatric pneumonia identified with LUS were not visible with radiograph.^3^ In the case presented here, significant morbidity may have been avoided had antibiotic therapy been initiated following the initial evaluation with LUS.

The advantages of LUS when compared to CXR extend beyond effectiveness and accuracy. Earlier detection of disease, reduced radiation exposure, and efficient bedside assessment are also clear advantages.^1^ ^,^ ^3^ ^–^ ^5^ In addition, LUS can be repeated at bedside to monitor disease progression or regression, allowing for informed ongoing treatment decisions. As point-of-care ultrasonography becomes an integral part of emergency medicine residency programs and standard of care, we hope to see more physicians trained to effectively perform, interpret, and clinically apply LUS, notably in the pediatric population.

This case presentation demonstrates the advantages of using LUS for clinical decision-making in the pediatric population. Early initiation of antibiotics based on LUS may help to avoid morbidity and mortality from treatment delay, and our case lends credence to the lingering question among emergency clinicians of whether to treat with antibiotics based on ultrasound findings that are discrepant with a CXR. Emergency clinicians should strongly consider prioritizing the findings of LUS in diagnosing and treating pediatric pneumonia, as well as support the training and dissemination of LUS as the superior modality to optimize care of this potentially vulnerable population.
